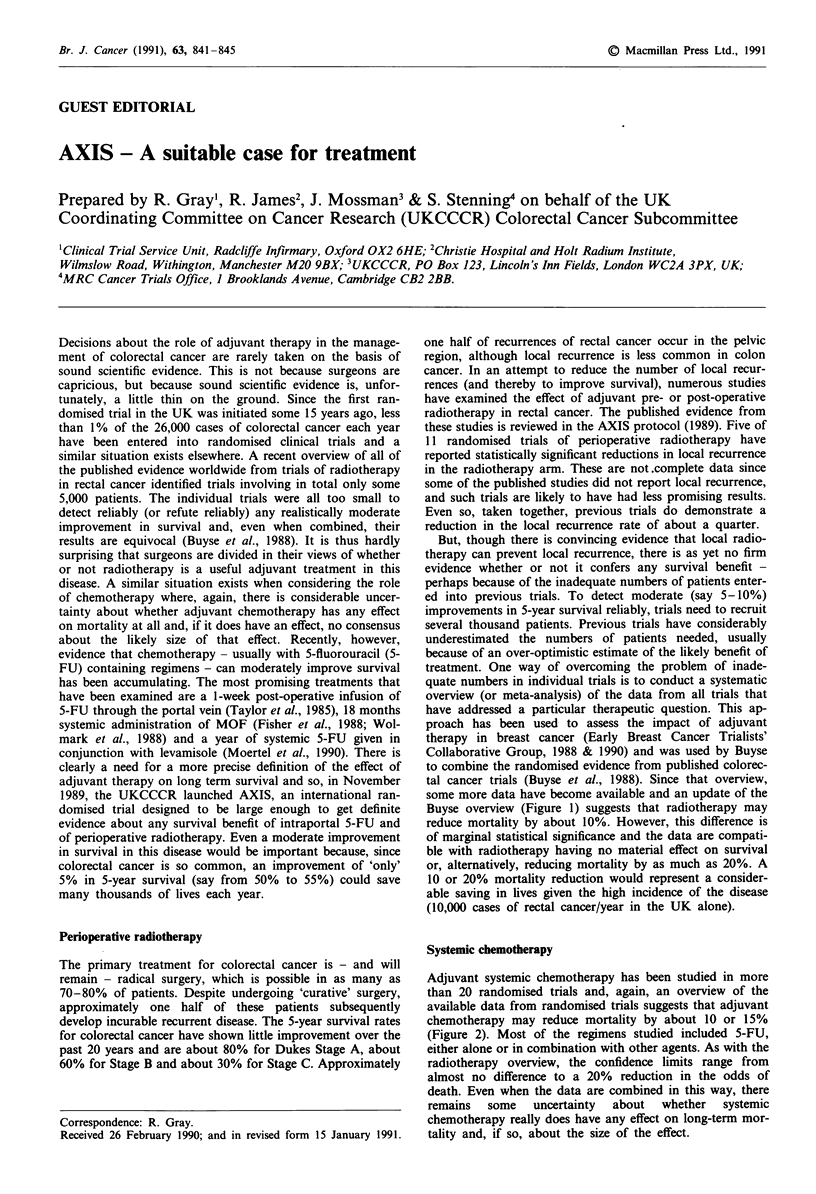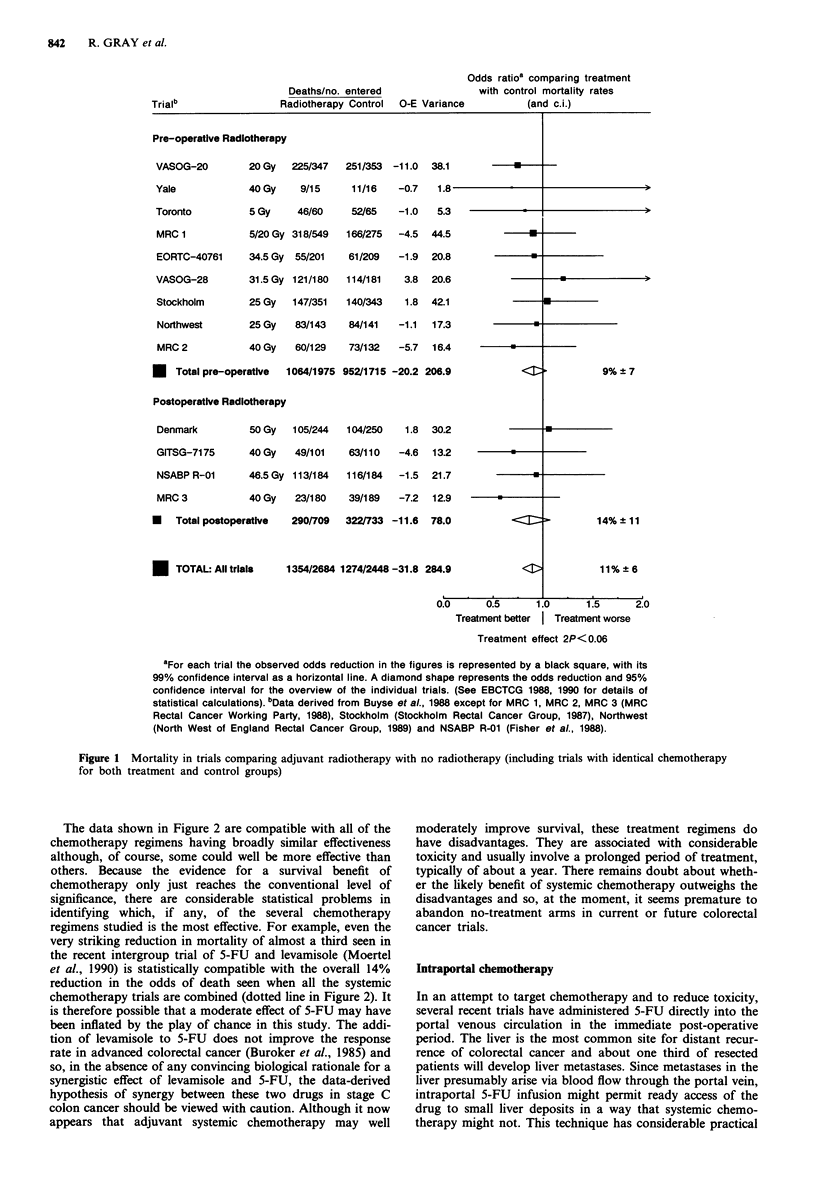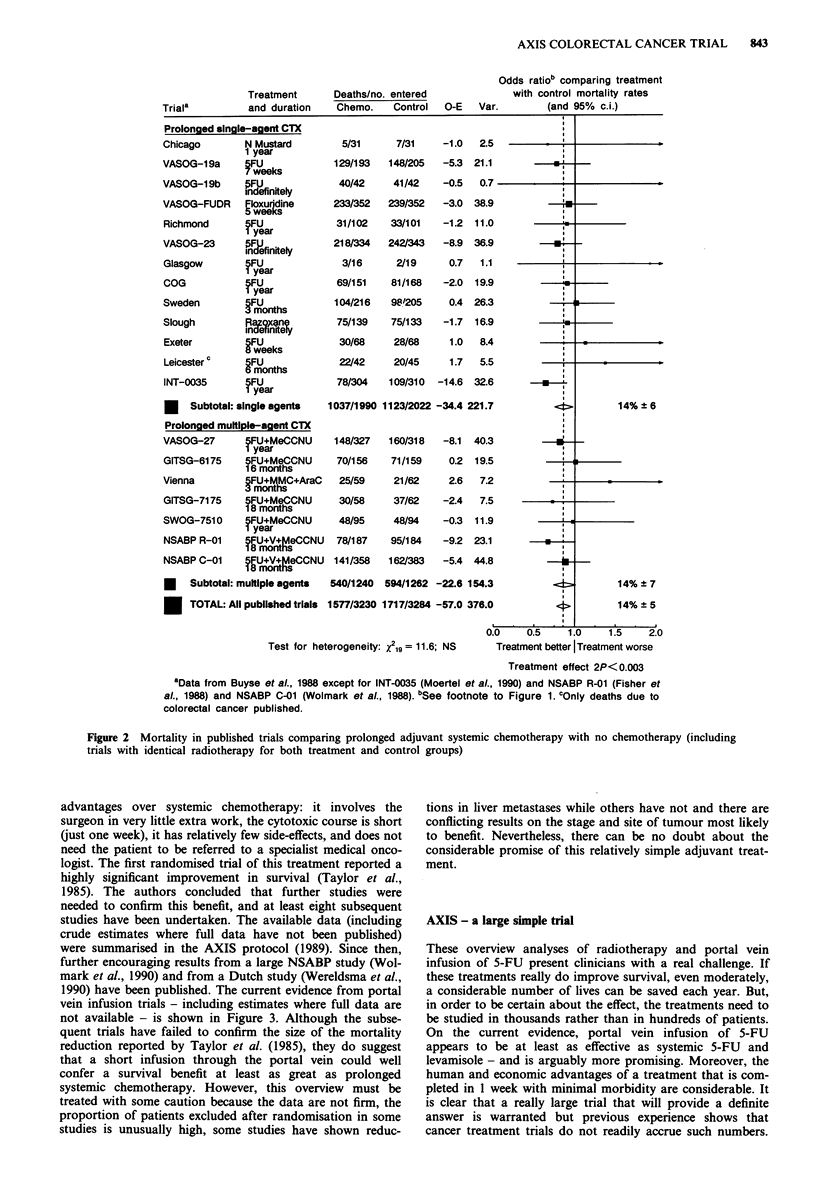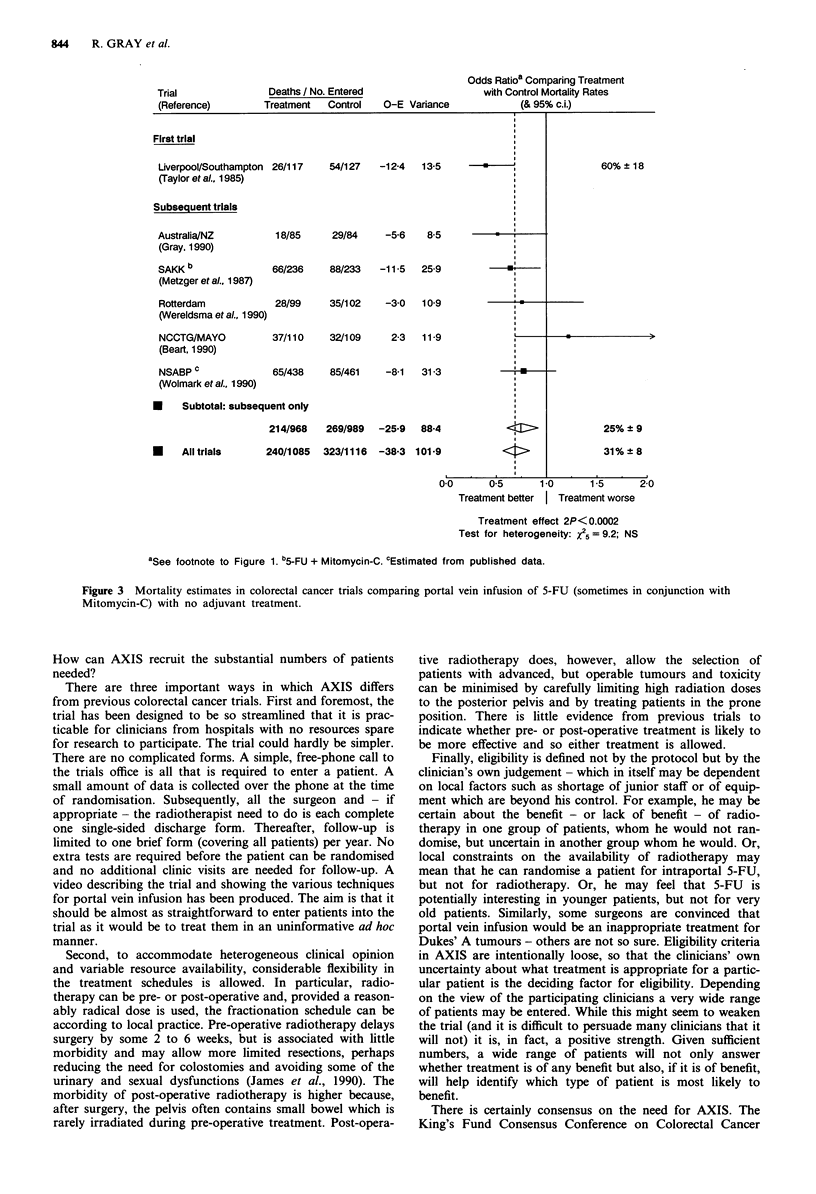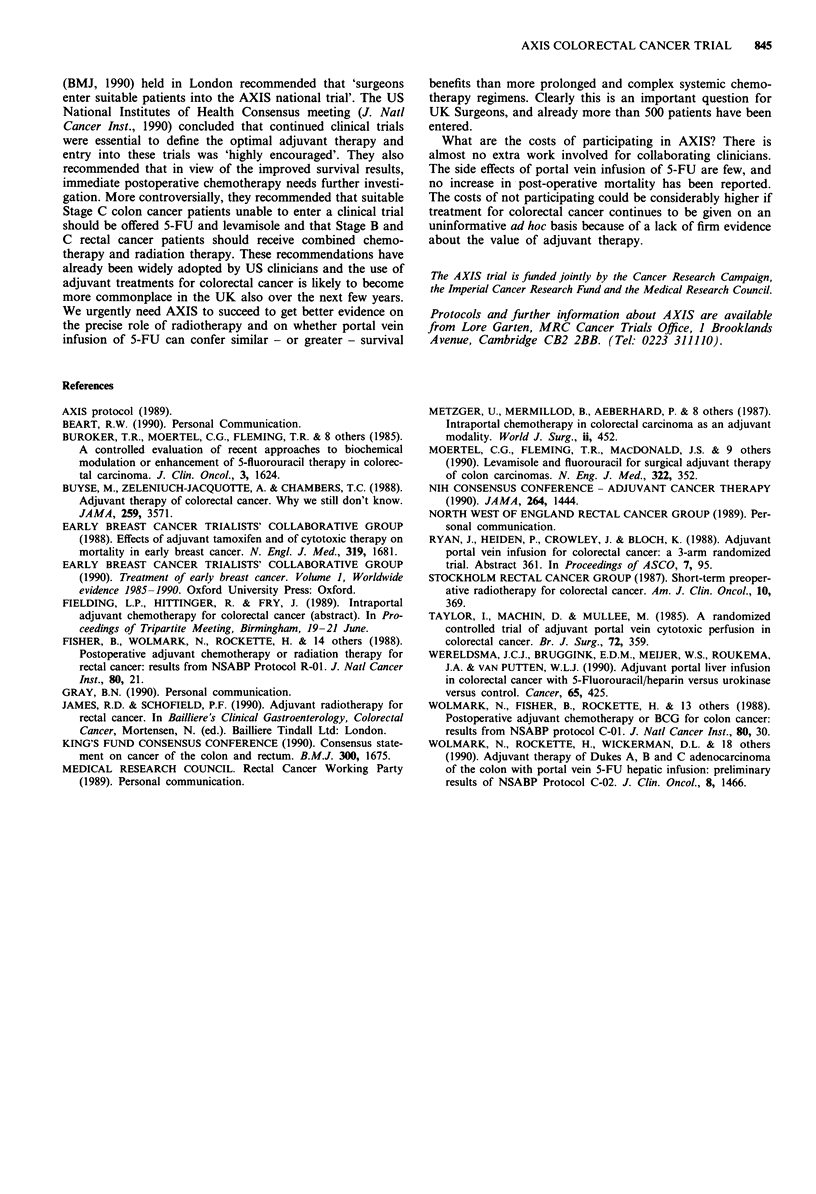# AXIS--a suitable case for treatment. UK Coordinating Committee on Cancer Research (UKCCCR) Colorectal Cancer Subcommittee.

**DOI:** 10.1038/bjc.1991.187

**Published:** 1991-06

**Authors:** R. Gray, R. James, J. Mossman, S. Stenning


Br. J. Cancer (1991), 63, 841-845 © Macmillan Press Ltd., 1991

GUEST EDITORIAL

### AXIS – A suitable case for treatment

Prepared by R. Gray[1], R. James[2], J. Mossman[3] & S. Stenning[4] on behalf of the UK Coordinating Committee on Cancer Research (UKCCCR) Colorectal Cancer Subcommittee

[1]*Clinical Trial Service Unit, Radcliffe Infirmary, Oxford OX2 6HE;* [2]*Christie Hospital and Holt Radium Institute, Wilmslow Road, Withington, Manchester M20 9BX;* [3]*UKCCCR, PO Box 123, Lincoln's Inn Fields, London WC2A 3PX, UK;* [4]*MRC Cancer Trials Office, 1 Brooklands Avenue, Cambridge CB2 2BB.*

Decisions about the role of adjuvant therapy in the management of colorectal cancer are rarely taken on the basis of sound scientific evidence. This is not because surgeons are capricious, but because sound scientific evidence is, unfortunately, a little thin on the ground. Since the first randomised trial in the UK was initiated some 15 years ago, less than 1% of the 26,000 cases of colorectal cancer each year have been entered into randomised clinical trials and a similar situation exists elsewhere. A recent overview of all of the published evidence worldwide from trials of radiotherapy in rectal cancer identified trials involving in total only some 5,000 patients. The individual trials were all too small to detect reliably (or refute reliably) any realistically moderate improvement in survival and, even when combined, their results are equivocal (Buyse *et al.*, 1988). It is thus hardly surprising that surgeons are divided in their views of whether or not radiotherapy is a useful adjuvant treatment in this disease. A similar situation exists when considering the role of chemotherapy where, again, there is considerable uncertainty about whether adjuvant chemotherapy has any effect on mortality at all and, if it does have an effect, no consensus about the likely size of that effect. Recently, however, evidence that chemotherapy – usually with 5-fluorouracil (5-FU) containing regimens – can moderately improve survival has been accumulating. The most promising treatments that have been examined are a 1-week post-operative infusion of 5-FU through the portal vein (Taylor *et al.*, 1985), 18 months systemic administration of MOF (Fisher *et al.*, 1988; Wolmark *et al.*, 1988) and a year of systemic 5-FU given in conjunction with levamisole (Moertel *et al.*, 1990). There is clearly a need for a more precise definition of the effect of adjuvant therapy on long term survival and so, in November 1989, the UKCCCR launched AXIS, an international randomised trial designed to be large enough to get definite evidence about any survival benefit of intraportal 5-FU and of perioperative radiotherapy. Even a moderate improvement in survival in this disease would be important because, since colorectal cancer is so common, an improvement of 'only' 5% in 5-year survival (say from 50% to 55%) could save many thousands of lives each year.

### Perioperative radiotherapy

The primary treatment for colorectal cancer is – and will remain – radical surgery, which is possible in as many as 70–80% of patients. Despite undergoing 'curative' surgery, approximately one half of these patients subsequently develop incurable recurrent disease. The 5-year survival rates for colorectal cancer have shown little improvement over the past 20 years and are about 80% for Dukes Stage A, about 60% for Stage B and about 30% for Stage C. Approximately one half of recurrences of rectal cancer occur in the pelvic region, although local recurrence is less common in colon cancer. In an attempt to reduce the number of local recurrences (and thereby to improve survival), numerous studies have examined the effect of adjuvant pre- or post-operative radiotherapy in rectal cancer. The published evidence from these studies is reviewed in the AXIS protocol (1989). Five of 11 randomised trials of perioperative radiotherapy have reported statistically significant reductions in local recurrence in the radiotherapy arm. These are not complete data since some of the published studies did not report local recurrence, and such trials are likely to have had less promising results. Even so, taken together, previous trials do demonstrate a reduction in the local recurrence rate of about a quarter.

But, though there is convincing evidence that local radiotherapy can prevent local recurrence, there is as yet no firm evidence whether or not it confers any survival benefit – perhaps because of the inadequate numbers of patients entered into previous trials. To detect moderate (say 5–10%) improvements in 5-year survival reliably, trials need to recruit several thousand patients. Previous trials have considerably underestimated the numbers of patients needed, usually because of an over-optimistic estimate of the likely benefit of treatment. One way of overcoming the problem of inadequate numbers in individual trials is to conduct a systematic overview (or meta-analysis) of the data from all trials that have addressed a particular therapeutic question. This approach has been used to assess the impact of adjuvant therapy in breast cancer (Early Breast Cancer Trialists' Collaborative Group, 1988 & 1990) and was used by Buyse to combine the randomised evidence from published colorectal cancer trials (Buyse *et al.*, 1988). Since that overview, some more data have become available and an update of the Buyse overview (Figure 1) suggests that radiotherapy may reduce mortality by about 10%. However, this difference is of marginal statistical significance and the data are compatible with radiotherapy having no material effect on survival or, alternatively, reducing mortality by as much as 20%. A 10 or 20% mortality reduction would represent a considerable saving in lives given the high incidence of the disease (10,000 cases of rectal cancer/year in the UK alone).

### Systemic chemotherapy

Adjuvant systemic chemotherapy has been studied in more than 20 randomised trials and, again, an overview of the available data from randomised trials suggests that adjuvant chemotherapy may reduce mortality by about 10 or 15% (Figure 2). Most of the regimens studied included 5-FU, either alone or in combination with other agents. As with the radiotherapy overview, the confidence limits range from almost no difference to a 20% reduction in the odds of death. Even when the data are combined in this way, there remains some uncertainty about whether systemic chemotherapy really does have any effect on long-term mortality and, if so, about the size of the effect.

Correspondence: R. Gray.
Received 26 February 1990; and in revised form 15 January 1991.

| Trial[b] | | Deaths/no. entered Radiotherapy | Control | O-E | Variance |
|---|---|---|---|---|---|
| **Pre-operative Radiotherapy** | | | | | |
| VASOG-20 | 20 Gy | 225/347 | 251/353 | -11.0 | 38.1 |
| Yale | 40 Gy | 9/15 | 11/16 | -0.7 | 1.8 |
| Toronto | 5 Gy | 46/60 | 52/65 | -1.0 | 5.3 |
| MRC 1 | 5/20 Gy | 318/549 | 166/275 | -4.5 | 44.5 |
| EORTC-40761 | 34.5 Gy | 55/201 | 61/209 | -1.9 | 20.8 |
| VASOG-28 | 31.5 Gy | 121/180 | 114/181 | 3.8 | 20.6 |
| Stockholm | 25 Gy | 147/351 | 140/343 | 1.8 | 42.1 |
| Northwest | 25 Gy | 83/143 | 84/141 | -1.1 | 17.3 |
| MRC 2 | 40 Gy | 60/129 | 73/132 | -5.7 | 16.4 |
| **Total pre-operative** | | **1064/1975** | **952/1715** | **-20.2** | **206.9** |
| **Postoperative Radiotherapy** | | | | | |
| Denmark | 50 Gy | 105/244 | 104/250 | 1.8 | 30.2 |
| GITSG-7175 | 40 Gy | 49/101 | 63/110 | -4.6 | 13.2 |
| NSABP R-01 | 46.5 Gy | 113/184 | 116/184 | -1.5 | 21.7 |
| MRC 3 | 40 Gy | 23/180 | 39/189 | -7.2 | 12.9 |
| **Total postoperative** | | **290/709** | **322/733** | **-11.6** | **78.0** |
| **TOTAL: All trials** | | **1354/2684** | **1274/2448** | **-31.8** | **284.9** |

[a]For each trial the observed odds reduction in the figures is represented by a black square, with its 99% confidence interval as a horizontal line. A diamond shape represents the odds reduction and 95% confidence interval for the overview of the individual trials. (See EBCTCG 1988, 1990 for details of statistical calculations). [b]Data derived from Buyse *et al.*, 1988 except for MRC 1, MRC 2, MRC 3 (MRC Rectal Cancer Working Party, 1988), Stockholm (Stockholm Rectal Cancer Group, 1987), Northwest (North West of England Rectal Cancer Group, 1989) and NSABP R-01 (Fisher *et al.*, 1988).

**Figure 1** Mortality in trials comparing adjuvant radiotherapy with no radiotherapy (including trials with identical chemotherapy for both treatment and control groups)

The data shown in Figure 2 are compatible with all of the chemotherapy regimens having broadly similar effectiveness although, of course, some could well be more effective than others. Because the evidence for a survival benefit of chemotherapy only just reaches the conventional level of significance, there are considerable statistical problems in identifying which, if any, of the several chemotherapy regimens studied is the most effective. For example, even the very striking reduction in mortality of almost a third seen in the recent intergroup trial of 5-FU and levamisole (Moertel *et al.*, 1990) is statistically compatible with the overall 14% reduction in the odds of death seen when all the systemic chemotherapy trials are combined (dotted line in Figure 2). It is therefore possible that a moderate effect of 5-FU may have been inflated by the play of chance in this study. The addition of levamisole to 5-FU does not improve the response rate in advanced colorectal cancer (Buroker *et al.*, 1985) and so, in the absence of any convincing biological rationale for a synergistic effect of levamisole and 5-FU, the data-derived hypothesis of synergy between these two drugs in stage C colon cancer should be viewed with caution. Although it now appears that adjuvant systemic chemotherapy may well moderately improve survival, these treatment regimens do have disadvantages. They are associated with considerable toxicity and usually involve a prolonged period of treatment, typically of about a year. There remains doubt about whether the likely benefit of systemic chemotherapy outweighs the disadvantages and so, at the moment, it seems premature to abandon no-treatment arms in current or future colorectal cancer trials.

### Intraportal chemotherapy

In an attempt to target chemotherapy and to reduce toxicity, several recent trials have administered 5-FU directly into the portal venous circulation in the immediate post-operative period. The liver is the most common site for distant recurrence of colorectal cancer and about one third of resected patients will develop liver metastases. Since metastases in the liver presumably arise via blood flow through the portal vein, intraportal 5-FU infusion might permit ready access of the drug to small liver deposits in a way that systemic chemotherapy might not. This technique has considerable practical

| Trial[a] | Treatment and duration | Deaths/no. entered Chemo. | Control | O-E | Var. | Odds ratio[b] comparing treatment with control mortality rates (and 95% c.i.) |
|---|---|---|---|---|---|---|
| **Prolonged single-agent CTX** | | | | | | |
| Chicago | N Mustard 1 year | 5/31 | 7/31 | -1.0 | 2.5 | |
| VASOG-19a | 5FU 7 weeks | 129/193 | 148/205 | -5.3 | 21.1 | |
| VASOG-19b | 5FU indefinitely | 40/42 | 41/42 | -0.5 | 0.7 | |
| VASOG-FUDR | Floxuridine 5 weeks | 233/352 | 239/352 | -3.0 | 38.9 | |
| Richmond | 5FU 1 year | 31/102 | 33/101 | -1.2 | 11.0 | |
| VASOG-23 | 5FU indefinitely | 218/334 | 242/343 | -8.9 | 36.9 | |
| Glasgow | 5FU 1 year | 3/16 | 2/19 | 0.7 | 1.1 | |
| COG | 5FU 1 year | 69/151 | 81/168 | -2.0 | 19.9 | |
| Sweden | 5FU 3 months | 104/216 | 98/205 | 0.4 | 26.3 | |
| Slough | Razoxane indefinitely | 75/139 | 75/133 | -1.7 | 16.9 | |
| Exeter | 5FU 8 weeks | 30/68 | 28/68 | 1.0 | 8.4 | |
| Leicester[c] | 5FU 6 months | 22/42 | 20/45 | 1.7 | 5.5 | |
| INT-0035 | 5FU 1 year | 78/304 | 109/310 | -14.6 | 32.6 | |
| **Subtotal: single agents** | | **1037/1990** | **1123/2022** | **-34.4** | **221.7** | **14% ± 6** |
| **Prolonged multiple-agent CTX** | | | | | | |
| VASOG-27 | 5FU+MeCCNU 1 year | 148/327 | 160/318 | -8.1 | 40.3 | |
| GITSG-6175 | 5FU+MeCCNU 16 months | 70/156 | 71/159 | 0.2 | 19.5 | |
| Vienna | 5FU+MMC+AraC 3 months | 25/59 | 21/62 | 2.6 | 7.2 | |
| GITSG-7175 | 5FU+MeCCNU 18 months | 30/58 | 37/62 | -2.4 | 7.5 | |
| SWOG-7510 | 5FU+MeCCNU 1 year | 48/95 | 48/94 | -0.3 | 11.9 | |
| NSABP R-01 | 5FU+V+MeCCNU 18 months | 78/187 | 95/184 | -9.2 | 23.1 | |
| NSABP C-01 | 5FU+V+MeCCNU 18 months | 141/358 | 162/383 | -5.4 | 44.8 | |
| **Subtotal: multiple agents** | | **540/1240** | **594/1262** | **-22.6** | **154.3** | **14% ± 7** |
| **TOTAL: All published trials** | | **1577/3230** | **1717/3284** | **-57.0** | **376.0** | **14% ± 5** |

Test for heterogeneity: $\chi^2_{19} = 11.6$; NS

Treatment effect $2P < 0.003$

[a]Data from Buyse *et al.*, 1988 except for INT-0035 (Moertel *et al.*, 1990) and NSABP R-01 (Fisher *et al.*, 1988) and NSABP C-01 (Wolmark *et al.*, 1988). [b]See footnote to Figure 1. [c]Only deaths due to colorectal cancer published.

**Figure 2** Mortality in published trials comparing prolonged adjuvant systemic chemotherapy with no chemotherapy (including trials with identical radiotherapy for both treatment and control groups)

advantages over systemic chemotherapy: it involves the surgeon in very little extra work, the cytotoxic course is short (just one week), it has relatively few side-effects, and does not need the patient to be referred to a specialist medical oncologist. The first randomised trial of this treatment reported a highly significant improvement in survival (Taylor *et al.*, 1985). The authors concluded that further studies were needed to confirm this benefit, and at least eight subsequent studies have been undertaken. The available data (including crude estimates where full data have not been published) were summarised in the AXIS protocol (1989). Since then, further encouraging results from a large NSABP study (Wolmark *et al.*, 1990) and from a Dutch study (Wereldsma *et al.*, 1990) have been published. The current evidence from portal vein infusion trials – including estimates where full data are not available – is shown in Figure 3. Although the subsequent trials have failed to confirm the size of the mortality reduction reported by Taylor *et al.* (1985), they do suggest that a short infusion through the portal vein could well confer a survival benefit at least as great as prolonged systemic chemotherapy. However, this overview must be treated with some caution because the data are not firm, the proportion of patients excluded after randomisation in some studies is unusually high, some studies have shown reductions in liver metastases while others have not and there are conflicting results on the stage and site of tumour most likely to benefit. Nevertheless, there can be no doubt about the considerable promise of this relatively simple adjuvant treatment.

### AXIS – a large simple trial

These overview analyses of radiotherapy and portal vein infusion of 5-FU present clinicians with a real challenge. If these treatments really do improve survival, even moderately, a considerable number of lives can be saved each year. But, in order to be certain about the effect, the treatments need to be studied in thousands rather than in hundreds of patients. On the current evidence, portal vein infusion of 5-FU appears to be at least as effective as systemic 5-FU and levamisole – and is arguably more promising. Moreover, the human and economic advantages of a treatment that is completed in 1 week with minimal morbidity are considerable. It is clear that a really large trial that will provide a definite answer is warranted but previous experience shows that cancer treatment trials do not readily accrue such numbers.

| Trial (Reference) | Deaths / No. Entered: Treatment | Deaths / No. Entered: Control | O−E | Variance | Odds Ratio[a] Comparing Treatment with Control Mortality Rates (& 95% c.i.) |
|---|---|---|---|---|---|
| **First trial** | | | | | |
| Liverpool/Southampton (Taylor et al., 1985) | 26/117 | 54/127 | −12·4 | 13·5 | 60% ± 18 |
| **Subsequent trials** | | | | | |
| Australia/NZ (Gray, 1990) | 18/85 | 29/84 | −5·6 | 8·5 | |
| SAKK [b] (Metzger et al., 1987) | 66/236 | 88/233 | −11·5 | 25·9 | |
| Rotterdam (Wereldsma et al., 1990) | 28/99 | 35/102 | −3·0 | 10·9 | |
| NCCTG/MAYO (Beart, 1990) | 37/110 | 32/109 | 2·3 | 11·9 | |
| NSABP [c] (Wolmark et al., 1990) | 65/438 | 85/461 | −8·1 | 31·3 | |
| **Subtotal: subsequent only** | **214/968** | **269/989** | **−25·9** | **88·4** | **25% ± 9** |
| **All trials** | **240/1085** | **323/1116** | **−38·3** | **101·9** | **31% ± 8** |

Treatment effect $2P < 0.0002$
Test for heterogeneity: $\chi^2_5 = 9.2$; NS

[a]See footnote to Figure 1. [b]5-FU + Mitomycin-C. [c]Estimated from published data.

**Figure 3** Mortality estimates in colorectal cancer trials comparing portal vein infusion of 5-FU (sometimes in conjunction with Mitomycin-C) with no adjuvant treatment.

How can AXIS recruit the substantial numbers of patients needed?

There are three important ways in which AXIS differs from previous colorectal cancer trials. First and foremost, the trial has been designed to be so streamlined that it is practicable for clinicians from hospitals with no resources spare for research to participate. The trial could hardly be simpler. There are no complicated forms. A simple, free-phone call to the trials office is all that is required to enter a patient. A small amount of data is collected over the phone at the time of randomisation. Subsequently, all the surgeon and – if appropriate – the radiotherapist need to do is each complete one single-sided discharge form. Thereafter, follow-up is limited to one brief form (covering all patients) per year. No extra tests are required before the patient can be randomised and no additional clinic visits are needed for follow-up. A video describing the trial and showing the various techniques for portal vein infusion has been produced. The aim is that it should be almost as straightforward to enter patients into the trial as it would be to treat them in an uninformative *ad hoc* manner.

Second, to accommodate heterogeneous clinical opinion and variable resource availability, considerable flexibility in the treatment schedules is allowed. In particular, radiotherapy can be pre- or post-operative and, provided a reasonably radical dose is used, the fractionation schedule can be according to local practice. Pre-operative radiotherapy delays surgery by some 2 to 6 weeks, but is associated with little morbidity and may allow more limited resections, perhaps reducing the need for colostomies and avoiding some of the urinary and sexual dysfunctions (James *et al.*, 1990). The morbidity of post-operative radiotherapy is higher because, after surgery, the pelvis often contains small bowel which is rarely irradiated during pre-operative treatment. Post-operative radiotherapy does, however, allow the selection of patients with advanced, but operable tumours and toxicity can be minimised by carefully limiting high radiation doses to the posterior pelvis and by treating patients in the prone position. There is little evidence from previous trials to indicate whether pre- or post-operative treatment is likely to be more effective and so either treatment is allowed.

Finally, eligibility is defined not by the protocol but by the clinician's own judgement – which in itself may be dependent on local factors such as shortage of junior staff or of equipment which are beyond his control. For example, he may be certain about the benefit – or lack of benefit – of radiotherapy in one group of patients, whom he would not randomise, but uncertain in another group whom he would. Or, local constraints on the availability of radiotherapy may mean that he can randomise a patient for intraportal 5-FU, but not for radiotherapy. Or, he may feel that 5-FU is potentially interesting in younger patients, but not for very old patients. Similarly, some surgeons are convinced that portal vein infusion would be an inappropriate treatment for Dukes' A tumours – others are not so sure. Eligibility criteria in AXIS are intentionally loose, so that the clinicians' own uncertainty about what treatment is appropriate for a particular patient is the deciding factor for eligibility. Depending on the view of the participating clinicians a very wide range of patients may be entered. While this might seem to weaken the trial (and it is difficult to persuade many clinicians that it will not) it is, in fact, a positive strength. Given sufficient numbers, a wide range of patients will not only answer whether treatment is of any benefit but also, if it is of benefit, will help identify which type of patient is most likely to benefit.

There is certainly consensus on the need for AXIS. The King's Fund Consensus Conference on Colorectal Cancer

(BMJ, 1990) held in London recommended that 'surgeons enter suitable patients into the AXIS national trial'. The US National Institutes of Health Consensus meeting (*J. Natl Cancer Inst.*, 1990) concluded that continued clinical trials were essential to define the optimal adjuvant therapy and entry into these trials was 'highly encouraged'. They also recommended that in view of the improved survival results, immediate postoperative chemotherapy needs further investigation. More controversially, they recommended that suitable Stage C colon cancer patients unable to enter a clinical trial should be offered 5-FU and levamisole and that Stage B and C rectal cancer patients should receive combined chemotherapy and radiation therapy. These recommendations have already been widely adopted by US clinicians and the use of adjuvant treatments for colorectal cancer is likely to become more commonplace in the UK also over the next few years. We urgently need AXIS to succeed to get better evidence on the precise role of radiotherapy and on whether portal vein infusion of 5-FU can confer similar – or greater – survival benefits than more prolonged and complex systemic chemotherapy regimens. Clearly this is an important question for UK Surgeons, and already more than 500 patients have been entered.

What are the costs of participating in AXIS? There is almost no extra work involved for collaborating clinicians. The side effects of portal vein infusion of 5-FU are few, and no increase in post-operative mortality has been reported. The costs of not participating could be considerably higher if treatment for colorectal cancer continues to be given on an uninformative *ad hoc* basis because of a lack of firm evidence about the value of adjuvant therapy.

*The AXIS trial is funded jointly by the Cancer Research Campaign, the Imperial Cancer Research Fund and the Medical Research Council.*

*Protocols and further information about AXIS are available from Lore Garten, MRC Cancer Trials Office, 1 Brooklands Avenue, Cambridge CB2 2BB. (Tel: 0223 311110).*

**References**

AXIS protocol (1989).

BEART, R.W. (1990). Personal Communication.

BUROKER, T.R., MOERTEL, C.G., FLEMING, T.R. & 8 others (1985). A controlled evaluation of recent approaches to biochemical modulation or enhancement of 5-fluorouracil therapy in colorectal carcinoma. *J. Clin. Oncol.*, **3**, 1624.

BUYSE, M., ZELENIUCH-JACQUOTTE, A. & CHAMBERS, T.C. (1988). Adjuvant therapy of colorectal cancer. Why we still don't know. *JAMA*, **259**, 3571.

EARLY BREAST CANCER TRIALISTS' COLLABORATIVE GROUP (1988). Effects of adjuvant tamoxifen and of cytotoxic therapy on mortality in early breast cancer. *N. Engl. J. Med.*, **319**, 1681.

EARLY BREAST CANCER TRIALISTS' COLLABORATIVE GROUP (1990). *Treatment of early breast cancer. Volume 1, Worldwide evidence 1985–1990*. Oxford University Press: Oxford.

FIELDING, L.P., HITTINGER, R. & FRY, J. (1989). Intraportal adjuvant chemotherapy for colorectal cancer (abstract). In *Proceedings of Tripartite Meeting, Birmingham, 19–21 June*.

FISHER, B., WOLMARK, N., ROCKETTE, H. & 14 others (1988). Postoperative adjuvant chemotherapy or radiation therapy for rectal cancer: results from NSABP Protocol R-01. *J. Natl Cancer Inst.*, **80**, 21.

GRAY, B.N. (1990). Personal communication.

JAMES, R.D. & SCHOFIELD, P.F. (1990). Adjuvant radiotherapy for rectal cancer. In *Bailliere's Clinical Gastroenterology, Colorectal Cancer*, Mortensen, N. (ed.). Bailliere Tindall Ltd: London.

KING'S FUND CONSENSUS CONFERENCE (1990). Consensus statement on cancer of the colon and rectum. *B.M.J.* **300**, 1675.

MEDICAL RESEARCH COUNCIL. Rectal Cancer Working Party (1989). Personal communication.

METZGER, U., MERMILLOD, B., AEBERHARD, P. & 8 others (1987). Intraportal chemotherapy in colorectal carcinoma as an adjuvant modality. *World J. Surg.*, **ii**, 452.

MOERTEL, C.G., FLEMING, T.R., MACDONALD, J.S. & 9 others (1990). Levamisole and fluorouracil for surgical adjuvant therapy of colon carcinomas. *N. Eng. J. Med.*, **322**, 352.

NIH CONSENSUS CONFERENCE – ADJUVANT CANCER THERAPY (1990). *JAMA*, **264**, 1444.

NORTH WEST OF ENGLAND RECTAL CANCER GROUP (1989). Personal communication.

RYAN, J., HEIDEN, P., CROWLEY, J. & BLOCH, K. (1988). Adjuvant portal vein infusion for colorectal cancer: a 3-arm randomized trial. Abstract 361. In *Proceedings of ASCO*, **7**, 95.

STOCKHOLM RECTAL CANCER GROUP (1987). Short-term preoperative radiotherapy for colorectal cancer. *Am. J. Clin. Oncol.*, **10**, 369.

TAYLOR, I., MACHIN, D. & MULLEE, M. (1985). A randomized controlled trial of adjuvant portal vein cytotoxic perfusion in colorectal cancer. *Br. J. Surg.*, **72**, 359.

WERELDSMA, J.C.J., BRUGGINK, E.D.M., MEIJER, W.S., ROUKEMA, J.A. & VAN PUTTEN, W.L.J. (1990). Adjuvant portal liver infusion in colorectal cancer with 5-Fluorouracil/heparin versus urokinase versus control. *Cancer*, **65**, 425.

WOLMARK, N., FISHER, B., ROCKETTE, H. & 13 others (1988). Postoperative adjuvant chemotherapy or BCG for colon cancer: results from NSABP protocol C-01. *J. Natl Cancer Inst.*, **80**, 30.

WOLMARK, N., ROCKETTE, H., WICKERMAN, D.L. & 18 others (1990). Adjuvant therapy of Dukes A, B and C adenocarcinoma of the colon with portal vein 5-FU hepatic infusion: preliminary results of NSABP Protocol C-02. *J. Clin. Oncol.*, **8**, 1466.